# Genetic and Molecular Approaches to Study Neuronal Migration in the Developing Cerebral Cortex

**DOI:** 10.3390/brainsci7050053

**Published:** 2017-05-05

**Authors:** Jacobus J. Dudok, Pim E. G. Leonards, Jan Wijnholds

**Affiliations:** 1 Department of Environment and Health, Vrije Universiteit Amsterdam, De Boelelaan 1087, Amsterdam 1081 HV, The Netherlands; pim.leonards@vu.nl; 2 Netherlands Institute for Neuroscience, Meibergdreef 47, Amsterdam 1105 BA, The Netherlands; j.wijnholds@lumc.nl; 3 Department of Ophthalmology, Leiden University Medical Center, Albinusdreef 2, Leiden 2300 RC, The Netherlands

**Keywords:** cortex, migration, neuron, progenitor, knockout, genetic, molecular

## Abstract

The migration of neuronal cells in the developing cerebral cortex is essential for proper development of the brain and brain networks. Disturbances in this process, due to genetic abnormalities or exogenous factors, leads to aberrant brain formation, brain network formation, and brain function. In the last decade, there has been extensive research in the field of neuronal migration. In this review, we describe different methods and approaches to assess and study neuronal migration in the developing cerebral cortex. First, we discuss several genetic methods, techniques and genetic models that have been used to study neuronal migration in the developing cortex. Second, we describe several molecular approaches to study aberrant neuronal migration in the cortex which can be used to elucidate the underlying mechanisms of neuronal migration. Finally, we describe model systems to investigate and assess the potential toxicity effect of prenatal exposure to environmental chemicals on proper brain formation and neuronal migration.

## 1. Introduction

During embryonic development, the central nervous system develops in a number of distinct steps. First, the chorda dorsalis instructs the ectoderm to form a neural fold, after which closure of this fold leads to the formation of the neural tube. Subsequently, the compartmentalization of the neural tube results in the formation of the prosencephalon, mesencephalon, and rhombencephalon which eventually develop into the forebrain, midbrain, and hindbrain, respectively. After the development of these different areas, the birth of neuronal cells begins, and these cells start migrating towards their proper position. This is performed in an “inside–outside” fashion that eventually results in a neocortex with six different and distinguishable layers. In the developing cortex, at the ventricular side of the cortex, there is a pool of progenitor cells where neurogenesis takes place. This area of neurogenesis is called the ventricular zone, and later during neurodevelopment basally of the ventricular zone, the subventricular zone forms. In these zones, progenitor cells undergo interkinetic nuclear migration (INM), meaning that their nuclei migrate basally and apically in this ventricular zone during their cell cycle [1,2]. Several apical localized proteins reside at the apical plasma membrane facing the future cortical ventricles. Removal of several of these proteins results in both a disrupted apical membrane, as well as affected cell division and aberrant neuronal migration [3].

In the 1960s, the first studies appeared in which neuronal migration in different model systems was investigated. During that period, research was performed using autoradiographic labelling and histological methods to assess the migration of neurons in rats and chicken embryos [4,5]. These studies were quickly complemented with in vitro studies of dorsal root ganglia tissue and organotypic cultures [6,7,8,9]. In the last 30 years, there has been extensive research on the process of neuronal migration, the molecular events that dictate this migration, aberrant neuronal migration due to genetic abnormalities, and the result of disturbance of this migration due to exogenous factors. Another major factor in the increase in the study of neuronal migration during the 1990s was the development of the mouse knockout technique, which allowed researchers to investigate the function of presumably essential genes in neuronal migration. Furthermore, the development of the in utero electroporation technique resulted in studies on the effects of downregulating genes required for proper migration on neuronal migration, and to allow these cells to express a fluorescent marker to follow the migration of cells with a downregulated gene over time. Finally, the recently developed Crispr-Cas9 technique will create new opportunities to study neuronal migration in further detail in vivo in animals, as well as ex vivo in cultured human induced pluripotent stem cell (iPSC) derived brain structures. In this review, we describe the latest developments in the field of neuronal migration and give an overview of the currently widely used different genetic and molecular approaches to study neuronal migration in the developing cortex. To achieve this, we first describe several widely-used animal models to study neuronal migration and then mouse knockouts with altered or aberrant neuronal migration. Thereafter, we elaborate on both the in utero electroporation technique, as well as the Crispr-Cas9 technique, and the new opportunities this will create in the study of neuronal migration. Finally, we discuss a number of models that can be used to assess the effect of exogenous factors—such as environmental chemicals—on brain development and neuronal migration.

## 2. Animal Models to Study Neuronal Migration

In the field of embryology and (neuro) development, there are many animal models which are widely used to study development. Some of these animal models are more appropriate in the use of molecular approaches for the study of neuronal migration, whereas other models are used to study neuronal migration using a genetic approach, or a combination of both a molecular as well as a genetic approach. In the field of neuronal migration, the most widely used animal model is the mouse. Although there are other animal models—including chicken embryos, zebrafish, and *C. elegans*—which are employed to study (brain) development, these are mostly used for more fundamental studies of (brain) development, and studying the effect of environmental chemicals and potential toxic compounds on brain development. The reason as to why mice are the most widely used animal model in this field of study is mostly due to the fact that genetic manipulation of the mouse has been used to study the involvement of several genes in neuronal migration, or to identify novel genes important for neuronal migration. Furthermore, in mice there are a number of mutant strains available which have aberrant neuronal migration. One of the best-known examples is the so-called reeler mouse, in which the *reelin* gene is defective [10,11,12]. Interestingly, this mouse has a very prominent cortical phenotype as the layering of the cortex is inverted [13]. Another related natural occurring mutant strain is the so-called scrambler mouse strain [14,15]. This mouse mutant displays a phenotype which is very comparable to the reeler mouse which was the result of aberrant splicing in the *mdab1* gene [16]. Although these mutant mouse strains (especially the reeler mutant mice) have been used to study the mechanisms behind neuronal migration in the pre-genetic recombination era, research in the field of neuronal migration increased significantly after the development and introduction of the genetic recombination technique in mice. In the next section, we describe the different genetic techniques, approaches, and genetically altered mouse models which have been used to study the mechanisms involved in neuronal migration and have contributed new information on these mechanisms.

## 3. Genetic Techniques

### 3.1. Classical Genetic Knockout and Knockin Mice

Since the development of the genetic recombination technique in mice in the 1980s and 1990s, it has been used to specifically delete a gene (“knockout”) to study the effects of its removal [17,18]. In the field of neuronal migration, this has led to the development of a number of mouse mutants with aberrant neuronal migration in the cortex. Knockout mice in which neuronal migration and layering are affected include the *p35*, *FGF-2*, *PS-1*, and *EMX2* [19,20,21,22]. Additionally, instead of specifically deleting a gene of interest, a subtle (point) mutation can be inserted in a specific gene of interest, the so-called gene-targeted knockin. This has been used in the *TrkB* gene, in which targeted mutation resulted in an altered TrkB protein where the Shc and PLCy docking sites are non-functional [23]. This is an elegant approach to complement a physiological knockout study, to shed more light on the underlying cellular and molecular mechanisms of a gene knockout mediated neuronal migration phenotype.

One disadvantage of a global knockout is that deleting a gene which has an essential role in early embryogenesis results in early lethality and therefore prevents studying the role of this gene in later embryogenesis processes. This can sometimes be circumvented by inactivating only one allele of a gene of interest, which in some mouse models still gives a clear phenotype, such as *Lis1* heterozygous knockout mice [24]. However, two other approaches are used to circumvent this problem: spatial, and/or temporal regulation of removal of a gene of interest.

### 3.2. Conditional Genetic Ablation: Spatial and Temporal Regulation

Using spatial regulation of gene removal, a gene can be specifically deleted in the cortex or the forebrain using the Cre/*loxP* conditional knockout technique. For this technique, two mouse lines are required, one in which the gene of interest (which should be ablated) is flanked by *loxP* recognition sites, and another in which the Cre recombinase expression is under the control of a promoter which specifically activates gene expression in the developing cortex. When crossing a conditional knockout mouse with a Cre reporter line, the gene is specifically and only removed in cells and tissue types in which the Cre recombinase gene is expressed. Cre reporter lines with expression of Cre in the developing cerebral cortex in neuroprogenitor cells (which are widely used to specifically delete a gene in these regions) include the Emx1-*Cre*, hGFAP-*Cre*, and Nestin-*Cre* reporter lines [25,26,27,28].

To study neuronal migration in the brain, the most widely used Cre reporter line is the Emx1-*Cre* line, as this line results in widespread expression of Cre protein specifically in the developing cortex and hippocampus, starting at approximately embryonic day 9.5. The *Cre* gene expression is under the control of the *Emx1* gene promoter, enhancers, and silencers resulting in a pattern of Cre expression comparable with the expression of Emx1 in cortical progenitor cells [27,28]. This Cre reporter line has been used in many studies to investigate the role of different proteins in the developing cortex and neuronal migration [29,30]. In the hGFAP-*Cre* line, *Cre* is under the expression of the human GFAP promoter, resulting in Cre expression widespread in the central nervous system, and expression of Cre starts at E13.5 in the forebrain [31]. In this Cre line, Cre is reported to be expressed in radial glial progenitor cells and their progeny, including neurons [32,33]. Using this Cre line revealed, for example, that loss of 14-3-3 proteins resulted in neuronal migration defects due to the loss of binding of Ndel1 proteins, and the addition of phosphomimetic Ndel1 proteins rescued the neuronal migration phenotype [34]. Furthermore, this Cre reporter line has been used in a number of other studies in which a gene was specifically removed in the central nervous system [35,36].

In the Nestin-*Cre* line, the *Cre* is expressed as a transgene under the rat Nestin promoter, resulting in Cre expression in both the central nervous system and in the peripheral nervous system. In this line Cre recombinase expression, as detected by the Cre recombinase mediated expression of ß-galactosidase, is observed from E15.5 onwards and this reporter line has been used to study the role of different genes in neuronal migration in the developing cortex [37,38,39,40,41,42].

Thus, these Cre reporter lines crossed with conditional knockout mice have been useful tools in specifically deleting a gene in the developing cortex, therefore allowing studies on the role of these genes, and circumvents the embryonic lethality observed in classical knockouts in which the gene is deleted in the germ line. One disadvantage, however, is that these lines display low levels of Cre expression in different cell types. Furthermore, in cells in which only temporarily (during embryonic development) the Cre is expressed, a floxed sequence will be removed. Therefore, one should always consider that an observed phenotype may be due to the fact that the gene of interest has been removed in other cell types than expected. Another important point is that, upon expression of Cre recombinase, it will take approximately 24–48 h before a protein is completely removed. Therefore, only 1–2 days after Cre expression starts a phenotype could be observed, which means that, for example, the Nestin-*Cre* line is not usable in the study of early embryonic processes as Cre expression in this line starts only at E15.5. Nevertheless, when considering these points, this approach is advantageous for study in an in vivo system of the effect of removing a gene on embryonic development and neuronal migration in the forebrain. In addition to conditional deletion of a gene of interest using the cre-*loxP* technique, there are also reporter lines which can inducibly express Cre to have spatial as well as temporal control of Cre expression. This can be achieved using the CreER technique, where Cre is fused to the estrogen receptor and application of taxomifen through the diet or an injection results in activation of Cre [43,44]. There are some inducible Cre mouse lines that can be used to temporally control Cre expression in the developing brain, such as the Nestin-*CreER* and GLAST-*CreER* lines; however, these are rarely used in studies of neuronal migration, and are most often used in genetic fate-mapping and lineage analysis studies [45,46].

### 3.3. Knockdown of Genes of Interest to Study the Effect on Neuronal Migration

The discovery of short interfering and short hairpin RNAs (siRNA and shRNA, respectively) has led to genetic knockdown strategies in the last decade to reduce the expression of a gene of interest by 50%–90% [47,48]. Initially, this strategy was used in in vitro experiments in cultured brain slices, or neuronal cultures where the siRNA oligonucleotides or shRNA constructs were transfected, after which these slices and neuronal cultures were used to study the effect of reducing the expression of a gene of interest [49,50]. In addition to in vitro expression by knockdown constructs, a conditional knockdown mouse could be made via injection of a knockdown expression construct where the promoter in front of the shRNA was disrupted by a *loxP*-stop-*loxP* cassette to create a transgenic mouse with an inactive promoter. The shRNA was then specifically and temporarily expressed in the developing cortex by crossing with one of the abovementioned Cre reporter lines. Caution should be taken with the so-called off-target effects of short hairpin RNA constructs. Therefore, it is of great importance to first assess the specificity of the used short hairpin RNA construct in vitro; and second, to have the proper controls, such as scrambled short hairpin RNA constructs. Additionally, multiple short hairpin RNA sequences should be used to obtain a similar phenotype. Still, there could be some variability in the extent to which the expression of a gene of interest is reduced, which can result in a variable phenotype, or variability in the penetrance of the phenotype. These are major disadvantages in using this technique in vivo in transgenic knockdown mouse models, therefore, although there are some transgenic floxed short hairpin mouse models available, these are not widely used in the study of neuronal migration [51,52]. In contrast, another elegant approach to express a shRNA or siRNA in the developing cortex in a subset of cells to achieve a knockdown of a gene of interest is by using in utero electroporation, which is described in full detail below.

### 3.4. In Utero Electroporation

As the name suggests, using the in utero electroporation technique during prenatal development, mouse embryos can be transfected with a genetic construct. With this technique, efficient transfer of genes in the developing mouse brain can be achieved [53]. A big advantage of this technique is that there is both temporal and spatial control of the transfection, which is achieved by positioning the pulse electrodes. In this way, in one embryonic brain (for example, in one side of the developing cortex), a transgenic knockdown construct is injected and electroporated, resulting in transfected progenitor cells. In contrast, the other side of the developing cortex is not transfected and is therefore unaffected, resulting in one embryonic cortex which has both an experimental condition as well as a control condition. A disadvantage and a point to consider, however, is the fact that short hairpin RNAs could have off-target effects and that there is a variability in the effect of different short hairpin RNAs. To control for this, one should use both scrambled short hairpin RNAs as a negative control and several short hairpin RNAs with different sequences against the target RNA to select the most efficient short hairpin RNA construct. Additionally, as there will be a variability in the efficiency of reducing expression of a target gene, different short hairpin RNAs should be used to investigate whether a comparable phenotype is observed. However, when these points are taken into account, using in utero electroporation of shRNAs against genes—which are expected to be important for neuronal migration—is an elegant method to study the functions of these genes in neuronal migration.

In several studies, the targeting construct contains both the shRNA against a gene of interest and a fluorescent protein, which has two advantages. First, cells in which knockdown has occurred can be identified relatively easily by the expression of the fluorescent protein. Second, the cells expressing the fluorescent protein can be used for lineage tracing to investigate whether the fluorescent cells (in which the expression of a gene of interest is reduced) behave differently or have another migration pattern than control cells in which the expression of the gene of interest is not downregulated by a shRNA. Additionally, time-lapse imaging can be used to follow and study the migration pattern and speed of fluorescently labelled cells. The method of using a fluorescent protein marker has been employed elegantly in several studies and revealed many genes which are important for proper neuron migration. Knockdown of these genes results in a population of fluorescent cells (which are also expressing the knockdown construct) that showed an aberrant migration pattern [54,55,56,57]. Additionally, this method can be used to transfect a developing cortex with fluorescent transcripts to mark a certain population of neural progenitor cells by using the proper promoter. This has been used to reveal N-cadherin expressing cells in the ventricular zone and the structure of these cells [58]. Furthermore, this method can be used to visualize morphology and track the migratory pattern of newborn neurons [54].

### 3.5. Crispr-Cas9

The Crispr-Cas9 gene editing tool will have a large impact in several research fields, including the field of neuroscience and the study of neuron migration [59]. This method can be used to quickly and relatively simply manipulate the genome, including the mouse genome [60]. As mice are widely used in the field of neuronal migration, it is expected that this methodology will become widely used in the coming years in this research field. Presently, the first studies using Crispr-Cas9 are emerging [61,62]. Crispr-Cas9 also allows non-homologous-end-joining (NHEJ) to generate gene and promoter insertion-deletion (indel) mutations, and homologous recombination (HR) to generate gene or promoter knockin, in human induced pluripotent stem cells (iPSCs). In combination with recent optimizations in differentiation protocols, this allows the study of neuronal migration and fate mapping in cultured human iPSCs-derived 3D brain structures including e.g., forebrain cortex [63,64,65,66,67]. The main advantage of this emerging technique is that it is relatively quick and easy for manipulations of the genome, both in vivo as in vitro, in cell lines. Although this method showed positive results in some studies, the reliability and efficiency of the Crispr-Cas9 technique in in utero electroporation experiments needs to be validated. Nonetheless, future neuronal migration studies could also employ Crispr-Cas9 gene editing studies in patient-derived iPSCs-derived brain structures which allow cellular and molecular mechanistic studies of human instead of animal diseased cortex, that would be advantageous in the further study of the mechanisms of neuronal migration and aberrations in a human model system.

## 4. Molecular Techniques

Several of the molecular techniques described here are used in genetic models or in combination with genetic techniques. A number of molecular techniques widely used in the field of studying neuronal migration include cell cycle analysis, morphology analysis, and migratory analysis of migration of newly born neuronal cells. Additionally, rescue experiments are performed on a knockout/knockdown basis to elucidate molecular mechanisms and pathways. Several of the abovementioned techniques are used in vivo; however, also in vitro (organotypic) cell culture models exist to study neuronal migration. The different molecular techniques employed in the field of studying neuronal migration are described in the following paragraphs.

### 4.1. Cell Cycle Analysis, Spindle Orientation, and Neuronal Migration

Regulation of cell division and the cell cycle in the ventricular and subventricular zone is very important in the birth of newly generated neuronal cells and the disturbance of these processes results in disrupted neuronal migration. Therefore, cell cycle analysis using a number of different molecular approaches is a standard technique used in the field of cortical development. First, the use of bromodeoxyuridine (BrdU) or ethynyldeoxyuridine (EdU) (an analog of thymidine and a marker for proliferative cells as it is incorporated in newly synthetized DNA in dividing cells) is straightforward in the detection of the amount of cell division which has occurred in a certain time window [68]. Injection of a pregnant mouse, or exposure of living tissue in vitro, for example with BrdU and sacrificing the mouse or fixing the in vitro tissue after a certain time, and subsequent detection of BrdU positive cells using an anti-BrdU immunostaining, revealed the amount of cell division which has taken place in this period. The time period for BrdU incorporation can be used to assess different parameters concerning cell cycle analysis and cell division. First, a short BrdU exposure, of 30 minutes for example, allows the analysis of the rate of cell division in this period to compare with a control and an experimental condition. Moreover, since a subpopulation of cells is labelled, this can also be used to investigate whether neuronal migration is affected in an experimental condition by comparing the position of the labelled cells in the cortex with the control condition. Normally, these dividing cells still reside in the subventricular zone; however, when neuronal migration is affected, these dividing cells show an aberrant localization pattern outside the subventricular zone. Second, a 24 h exposure to BrdU can be used in combination with anti-Ki67 immunostaining (which is a marker for dividing cells) to determine the population of cells which have exited the cell cycle [69,70]. The population of cells (which are BrdU+ and Ki67- after a 24 h exposure to BrdU) as a percentage of the total pool of BrdU+ cells are the cells which have exited the cell cycle. Again, this could be used to determine whether more cells reside in the subventricular zone in an experimental condition to indicate a defective or affected neuronal migration. Finally, prenatal exposure to BrdU and postnatal analysis of cortical layering could be used to investigate whether progenitor cells (which are born in a certain timeframe) migrated to the proper location in the cortex. There are several genetic mouse models with an aberrant neuron migration which also show a disturbed cell cycle and cell division in the developing cortex, which has been shown using the above-mentioned methods [20,29,71,72,73].

In addition to cell cycle analysis and rate of division analysis, a widely-used method in studying potential cortical migration abnormalities is the analysis of spindle orientation in mitotic cells in the ventricular zone. Using phospho-histone H3 immunostaining—a marker for mitotic cells—the orientation of the mitotic spindle can also be determined. Vertical orientation of the spindle and cleavage results in symmetric division, whereas horizontal orientation and cleavage of the spindle results in asymmetric division of progenitor cells [74]. Disturbances of spindle orientation and mode of division during cortical development affects the pool of progenitor cells and the different types of neuronal cells which are born from these progenitor cells. Abnormalities in spindle orientation in most cases are associated with disturbed neuronal migration, making it worthwhile to investigate spindle orientation when neuronal migration defects are observed in a (genetic) mutant [75,76,77]. A main advantage of these methods is that they are relatively quick, easy and straightforward in assessing whether cell cycle and spindle orientation is affected upon removal of a gene of interest in a mouse model. However, it is important to mention that cell cycle analysis in progenitor cells is related to neuronal migration, but is not a direct measure for neuronal migration. Additionally, cell cycle analysis is an indication for progenitor and neural stem cell behavior. Therefore, abnormalities in neuronal migration in mouse mutants with an aberrant cell cycle may reflect secondary effects.

### 4.2. Centrosome Position and Function, Cytoskeletal Dynamics, and Time-Lapse Imaging

In addition to cell cycle analysis and spindle orientation, the position and functioning of the centrosome are directly associated with neuronal migration [78,79]. During cortical development, the progenitor cells undergo INM, meaning that their nuclei migrate basally and apically in this ventricular zone during their cell cycle, which is crucial for proper brain development [1,2]. In order for INM to function properly, both a centrosome and a leading process is required. The centrosome is the site where the microtubules emanate, and is positioned just in front of the nucleus. The leading process extends to the basal site of the developing cortex and is stabilized by microtubules, along which the nucleus can migrate. As both the functioning of the centrosome and the leading process are essential for neuronal migration and a direct measure for neuronal migration, it is important to study the functioning of the centrosome, cytoskeletal dynamics, and the leading process. Centrosome function can be studied by using a fluorescent marker for centrosomes combined with time-lapse imaging [80]. Time-lapse imaging can be performed in cortical tissue (which is obtained after in utero electroporation of gene constructs) to knockdown a gene of interest and to coexpress a fluorescent marker in these cells. Using this approach, the INM of progenitor cells can be investigated over longer time periods of hours, thus allowing study of the dynamics of neuronal migration. Using this method, the leading process can also be studied, as shown by the example in [81]. Additionally, using fluorescent microtubule markers such as EB3, both microtubule and cytoskeletal dynamics and the dynamics of INM along these microtubules can be studied. This has been done in the study of Tsai et al., in which the roles of LIS1 and dynein have been elegantly shown in neuronal migration in live brain tissue using time-lapse imaging [82]. In another study using this method, it was shown that microtubule stabilization at the leading process tip is essential for centrosomal movement and neuronal migration [83]. The main advantages of these methods is that they can be used to show a direct effect on neuronal migration of removing or knocking down a gene of interest, and that time-lapse imaging can be used to study the dynamics of this neuronal migration. A disadvantage is that both in utero electroporation and subsequent time-lapse imaging of neuronal migration are relatively difficult techniques for which both considerable time and experience is required in order to properly perform these experiments.

### 4.3. Techniques to Study Tangential Neuronal Migration

In contrast to radial migration, where progenitor cells migrate from their apical position in the ventricular and subventricular zones towards more basal positions, there is also tangential neuronal migration that occurs in the developing brain. In tangential migration, neurons migrate within the developing brain from one region to another, e.g., interneurons from the ganglionic eminence towards the cerebral cortex [84]. Several of the already mentioned methods and techniques can also be used to study tangential neuronal migration. As these cells are generated in regions other than their definitive position, lineage-tracing is a suitable technique to investigate the original area of cell generation and definitive location [85]. Additionally, viral mediated labeling with fluorescent markers specifically expressed in tangentially migrating progenitor cell populations (in slice cultures) combined with time-lapse imaging, is another suitable method that can be used to investigate and track tangential migration patterns [86,87]. A third method suitable for the study of tangential migration is a focal electroporation technique used to obtain the local electroporation of a fluorescent marker in the ganglionic eminence, which cultures these slices to follow the migration pattern of tangential migration cells [88]. This technique has been used to reveal novel migratory streams of interneurons [88]. Together, these above-mentioned techniques can be used to study tangential migration and migratory patterns.

### 4.4. Cortical Layer Neuronal Cell Population Markers

There are several markers available that can label the specific population of neuronal cells, and/or the population of neuronal cells in specific cortical layers. Immunohistochemical analysis of cortical tissue (both prenatal and postnatal), can reveal whether the different neuronal cell types are in their appropriate layer, or whether their localization has been disturbed (possibly due to neuronal migration defects). Widely-used cortical layer markers include Ctip2 and Cux1, which are markers for early-born layer V–VI and late-born layer II–IV neurons, respectively [89,90,91]. Other cortical layer markers include Cux2 and Lhx2, which are markers for layers II/III to IV; Brn2, a marker for layer II/III and V; Rorß, which marks layer IV; Opn3, which is a marker for layer V; and FoxP2, which is a marker for layer VI [91]. In the developing cortex, Pax6, Tuj1, and Tbr1 are widely-used markers for marking radial glial cells and neuronal cells, respectively [92,93,94,95]. In addition to the immunostaining of these different neuron populations, one can also employ in situ hybridization techniques to detect different neuronal populations and their distribution in the brain. The use of these markers is a relatively easy and straightforward method to quickly determine whether cortical layering and organization has been affected, and can be used as a first step in detecting potential neuronal migration abnormalities in (mouse) model systems.

### 4.5. In Vitro Organotypic Cultures

Although most studies and techniques used to investigate neuronal migration are performed in fixed cortical tissue, there are some in vitro models that can be used to study neuron migration. The large advantage of in vitro models is that they are easier to manipulate and can be used for live cell imaging of neuron migration. In this field of study, the most widely used in vitro model system are organotypic slice cultures [96,97]. These cultures can be used to study the effect of (pharmacological) compounds on neuronal migration [98]. Organotypic cultures can be exposed to these compounds globally, but local injection in the slice is also used [99]. Additionally, organotypic slices can be used for so-called ex vivo electroporation to overexpress a protein, express a fluorescent protein, or express a knockdown construct [100,101]. Recently, the first live cell imaging strategies of cortical explant cultures have been described [102]. The main advantage is that a dynamic view on neuronal migration can be acquired, and possible alterations thereon upon knockdown of genes of interest.

## 5. Neurotoxicology Models to Study Neuronal Migration

In the field of toxicology, the effect of prenatal exposure to environmental chemicals and potential toxic compounds on fetal brain development has been widely studied, and there are many model systems used in this field of study. First, the zebrafish is the most widely used model system to assess the potential toxic effect of environmental chemicals on neurodevelopment [103]. Although zebrafish brain development is different from mammalian brain development, zebrafish can still be exploited to study neuron migration [104]. Additionally, zebrafish can be used to relatively quickly screen a wide array of potential neurotoxic compounds, where identified compounds (with a clear effect on brain development and neuronal migration) can be used for further screening in mammalian model systems. Another advantage to zebrafish is that there are simple behavioral assays available to select exposed zebrafish that show aberrant behavior and to investigate whether brain development and/or neuronal migration has been affected [105]. The other model system used to study the potential disruptive effect of environmental chemicals on neurodevelopment are rodents. Prenatal exposure to environmental chemicals, ethanol or other compounds (where it is expected that they might interfere with proper development), is conducted to study the effect of these compounds on fetal brain development [106,107,108]. As an example, rats exposed to the environmental contaminant polychlorinated biphenyls (PCBs) [109], or methylmercury [110] showed disruptions in the neuronal migration in the cerebral cortex. Aside from the radial neuronal migration disruption by PCBs in the fetal cortex, the neuronal progenitor cell cycle exit was also disrupted [109], which was not found with methylmercury [110]. The PCB neuronal migration effects were only observed in the caudal region, while the cell cycle effects occurred in the central region. It is possible that the thyroid hormone (TH) axis was involved in this process by action on the actin cytoskeleton [109]. Actin cytoskeleton plays an important role in neuronal migration [111]. Actin polymerization is dependent on thyroid hormones T_4_ and rT_3_, and acts by anchoring guidance molecules used for neuronal migration [112,113]. It is known that PCBs, especially the hydroxy metabolites of PCBs, can competitively bind to the TH transport protein transthyretin (TTR) which can affect the TH axis [114,115]. A wide range of environmental contaminants have been shown to disrupt the TH system [116,117]; an overview of mechanism-based in vitro testing approaches for TH disruption can be found in [118].

In addition to these models, organotypic cultures can also be used to study the effect of environmental chemicals on these processes [119]. These models bridge the gap between receptors and the brain, and can be used to screen neurotoxic compounds. Furthermore, in vitro models can be used to study the effects of toxicants on neuron viability, differentiation, migration, and neuronal signaling. For example, fetal rat cortical cells were used to study the neurotoxic effects of polybrominated diphenyl ethers (PBDEs) on protein expression in the formation of the cytoskeleton involved in cell migration [120]. Low doses of a PBDE (BDE-99) showed changes in the cytoskeleton proteins. In another in vitro study with PBDEs using human neural progenitor cells, a decreased migration of these cells and reductions in the differentiation into neurons and oligodendrocytes were observed [121]. Interestingly, co-exposure with TH T_3_ (triiodothyronine) resulted in a reduction of the migration effects; demonstrating again the importance of the TH axis in relation to neuronal migration and the cytoskeleton formation.

## 6. Conclusions and Future Developments

There are several elegant genetic and molecular techniques which have been developed and used in the last two decades to study neuronal migration in the developing cortex. This has revealed a number of genes and genetic pathways important in regulating neuron migration and elucidated several underlying mechanisms in neuronal migration. When suspecting a defective neuronal migration in a new genetic mouse model for example, there are a number of steps which can be undertaken to show that there is an altered neuronal migration and to further elucidate the underlying mechanism. The application of molecular approaches can be used whether there is a mislocalization of neuronal cell types in the cortex. Additionally, methods to investigate the cell cycle and cell division will reveal whether this is affected. To further elucidate the molecular mechanism, one could make use of a combination of genetic and molecular techniques by transfecting rescue constructs on a knockout background or knockdown constructs on a control background combined with a fluorescent marker. These are a number of approaches which are part of a standard repertoire to investigate neuronal migration.

It is expected that the recently developed Crispr-Cas9 in combination with human iPSCs-derived cerebral organoids techniques will open up a wealth of new possibilities in the field of studying brain development and neuronal migration. Moreover, the first studies in which human fetal brain tissue was used recently appeared [122]. In the coming decades, neuronal migration and cortical development can be studied in more detail and will further reveal several mechanisms in early cortical development, generation of neuronal populations, and neuronal migration to their proper final destination.
